# Treatment of Simple Cavities

**Published:** 1869-03

**Authors:** W. H. Waite

**Affiliations:** Liverpool, England.


### SELECTED ARTICLES.

### ARTICLE VI.

### *Treatment of Simple Cavities.*

BY W. H. WAITE, D D.S., LIVERPOOL, ENGLAND.

By "Simple Cavities" we understand those only which involve no encroachment on the pulp cavity of the tooth. They may occur in every conceivable situation, but are most commonly found upon the approximal surfaces of front teeth, and the masticating surfaces of back teeth. For successful treatment, three principal considerations are essential.

1st. Freedom of access. This is to be obtained by wedging, either with india rubber or wood, the latter being probably better of the two, or by filing, or the use of the chisel, according to the position and nature of the cavity. Access should be clear enough to permit the operator to see every portion of the cavity, either directly or with the mirror, and also to allow the excavator and plugger touching the whole floor and sides of the cavity to any extent which may be desired. Whatever is necessary to afford such access ought not to be spared, either out of respect to the appearance of the tooth or the feelings of the patient—since if the cavity is worth filling at all, it is certainly worth doing in the best possible manner.

2ndly. Strong Walls. These are most difficult to secure in front teeth, and it is often necessary to sacrifie some amount of substance in order to obtain sufficient strength for thoroughly condensing the gold against. If this point be overlooked in preparing the cavity it is pretty sure to thrust its importance forward upon the operator's attention during the introduction of the gold, probably by giving way of the outer or inner wall of the enamel, disfiguring the contour of the tooth, and rendering the operation highly unsatisfactory to operator and patient.

3rdly. Good retaining points. If adhesive gold is employed, these should consists of two or more small holes drilled in opposites sides of the cavity, and they should be filled as solidly as possible. If non-adhesive gold is used, the shape of the cavity should be such as securely to retain the filling after its introduction. In approximal cavities the sides should be parallel, and the upper and the lower surfaces slightly undercut. In the cylindrical cavities the walls should be as nearly even as possible, slightly undercut beneath the enamel.

These precautions are indispensible to proper preparation of simple cavities ; in these we most frequently come upon sensitive dentine, the remedies for which are, creosote and tannin, creosote and chalk, chloride of zinc, &c., &c.

Time, labour and care spent in forming the cavity, will be repaid with interest, by facility of introducing the filling, and by the character of the operation when completed.—*Canada Journal of Dental Science.*

---

